# Multi-Regional Pelvic Floor Muscle Function Diagnosis System Based on Inflatable Stretchable Electrode Array

**DOI:** 10.3390/healthcare12191910

**Published:** 2024-09-24

**Authors:** Hailu Chen, Siming Wu, Yinfeng Wang, Yinjuan Chang, Mingjie Li, Zhenwei Xie, Shengming Wang

**Affiliations:** 1 Polytechnic Institute of Zhejiang University, Zhejiang University, Hangzhou 310015, China; 22060079@zju.edu.cn; 2 Department of Pediatrics and Adolescent Gynecology, The Children’s Hospital, Zhejiang University School of Medicine, National Clinical Research Center for Child Health, Hangzhou 310057, China; 22118429@zju.edu.cn; 3 Department of Gynecology, Women’s Hospital, Zhejiang University School of Medicine, Hangzhou 310006, China; wangyf1004@zju.edu.cn (Y.W.); chang6416886@zju.edu.cn (Y.C.); xiezw@zju.edu.cn (Z.X.); 4 Key Laboratory of Advanced Micro/Nano Electronic Devices & Smart Systems of Zhejiang, College of Information Science and Electronic Engineering, Zhejiang University, Hangzhou 310027, China; 22331067@zju.edu.cn; 5 Zhejiang University-University of Edinburgh Institute, Zhejiang University, Haining 314499, China

**Keywords:** functional diagnosis technology, regional muscle contribution assessment, precise location of abnormal muscles, stretchable electrode array probe, pelvic floor muscle

## Abstract

Background: Effective prevention and treatment of pelvic floor dysfunction (PFD) necessitates the identification of lesions within the complex pelvic floor muscle (PFM) groups associated with various symptoms. Here, we developed a multi-region pelvic floor muscle functional diagnosis system (MPDS) based on an inflatable stretchable electrode array, which aids in accurately locating areas related to PFD. Methods: Clinical diagnostic experiments were conducted on 56 patients with postpartum stress urinary incontinence (PSUI) and 73 postpartum asymptomatic controls. MPDS collects pelvic floor electromyography from all participants. By assessing EMG parameters such as activation time differences (ATD) and using Jensen–Shannon (JS) divergence to verify, with the aim of locating target muscle groups with functional abnormalities. Results: Clinical test results showed that by observing the AT sequence of the PSUI group and the control group, muscle groups with functional abnormalities in the Pubococcygeus muscle (PC) and Puborectalis muscle (PR) regions could be preliminarily diagnosed. In the assessment of regional muscle contribution values based on JS divergence, it was verified that the contribution values of rapid contraction in the PC and PR regions of the PSUI group were relatively lower compared to those of the control group, which correlated with urinary control dysfunction. Conclusions: These experiments demonstrate that the MPDS helps in accurately locating target muscle groups with functional abnormalities, showcasing its potential in precise assessment of complex muscle groups such as PFM, which may improve diagnostic precision and reliability.

## 1. Introduction

Pelvic floor dysfunction (PFD) denotes a spectrum of disorders resulting from defects, degeneration, injury, and functional impairments of the pelvic floor support structures [1]. This spectrum encompasses pelvic organ prolapse, urinary incontinence, defecation disorders, sexual dysfunction, and pelvic pain, among other conditions that seriously affect physical and mental health [2,3,4]. Pregnancy, childbirth, and menopausal aging stand as primary etiological factors [5].

The pelvic floor muscles (PFM) are structurally complex and finely specialized [6], consisting of different small muscle groups in the outer, middle, and inner layers. Different symptoms of PFD correlate with specific deficiencies in PFM function [7]. Therefore, achieving precise assessment of PFM dysfunction areas can aid in clinical diagnosis and targeted treatment.

Surface electromyography (sEMG) of the PFM stands as the main technology for assessing PFM function [8,9,10,11,12,13]. This technique involves the insertion of electrodes into the vagina or rectum to collect signals, offering advantages such as non-invasiveness, simplicity, and repeatability [14,15,16]. Currently, dual-channel rigid vaginal surface electrodes have been widely employed in clinical settings to aid in the diagnosis of pelvic floor disorders [14]. Polo proposed the implementation of a portable electromyography prototype for detecting fatigue in pelvic floor muscles and others [17]. Koenig introduced an approach utilizing sEMG signal acquisition to assess pelvic floor muscle activity in individuals with and without urinary incontinence [18]. Dias proposed an evaluation method based on high-density electrode arrays to assess pelvic floor hyperactivity symptoms in women with bladder pain syndrome [19].

However, existing technologies inadequately consider the complex structure of the PFM. They either treat the PFM as a whole entity or mechanically divide them into regions, thus failing to obtain detailed information about specific muscles [14,20]. Consequently, there is a lack of in-depth research on the muscles in different regions of the pelvic floor, making it impossible to express the dynamic compensatory relationships between muscles in various disease regions during the evaluation process or the involvement of local muscles in overall contraction [13,14,21,22]. Therefore, an increasing number of studies are focusing on the anatomical and functional zoning of the pelvic floor. Lin discovered risk factors for PFD using 3D ultrasound [23]. You used magnetic resonance imaging to predict postpartum stress urinary incontinence (PSUI) [24]. Although these studies have explored regional PFD, they suffer from drawbacks such as bulky examination equipment, complex operation, and high subjectivity, making them difficult to widely apply in clinical diagnosis. Precisely locating specific dysfunctional muscles and implementing highly targeted interventions are key to the accurate diagnosis and treatment of PFD, but they also present significant challenges in pathological research.

Therefore, the paper proposes a multi-region pelvic floor muscle functional diagnosis system (MPDS) based on an early-developed airbag-type stretchable electrode array (ASEA) [25,26] as the foundation for the detection device. This system collects EMG from patients with PSUI and individuals who are asymptomatic postpartum. By assessing electromyographic parameters such as activation time differences (ATD) and rapid contraction amplitude (RCP), we identify regions of localized pelvic floor muscle dysfunction. Further confirmation of these abnormal regions is supported through the comparison of regional contribution based on Jensen–Shannon (JS) divergence [27] and 3D mapping of feature parameters, with the aim of improving diagnostic accuracy and reliability. Clinical trial results demonstrate that this system can effectively evaluate differences in muscle compensatory effects among populations and identify the causes of abnormal states at the level of regional muscle synergy, aiding in the personalized and precise selection of treatment sites. To validate the assessment performance of this system in clinical applications, clinical experiments were conducted using the MPDS for PFM coordination and kinetic assessment. A total of 57 patients with PSUI and 73 healthy controls participated in the experiment. Participants performed a series of sustained standard contraction exercises as well as explosive standard contraction exercises. Precise evaluations of muscle groups in PFM regions dominated by slow-twitch and fast-twitch muscles were conducted to assess related disease conditions and abnormal functional areas. Clinical test results showed that by observing the AT sequence of the PSUI group and the control group, muscle groups with functional abnormalities in the PC and PR regions could be preliminarily diagnosed. Furthermore, in the assessment of regional muscle contribution values based on JS divergence, it was verified that the contribution values of rapid contraction in the PC and PR regions of the PSUI group were relatively lower compared to those of the control group, which correlated with urinary control dysfunction. These experiments demonstrate that the MPDS helps in accurately locating target muscle groups with functional abnormalities, showcasing its potential in precise assessment of complex muscle groups.

## 2. Materials and Methods

### 2.1. The Collection Probe and Regional Division in MPDS

**Figure 1:**
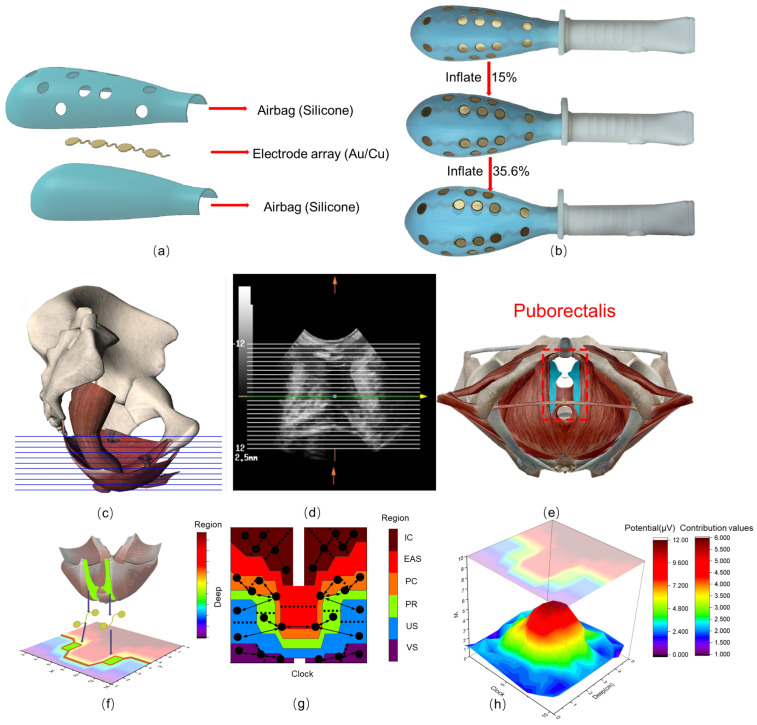
ASEA probe and the concept of regional division based on physiology. (**a**) Structural diagram of ASEA and (**b**) physical diagram; (**c**) side view schematic of the TUI imaging process, taking TI images at a distance of 2.5 mm; (**d**) spatial relationship of the slices represented in the entire panel reconstructed from the corresponding TUI images in, the green arrows and lines represent the horizontal axes separated by equal distances, and the yellow arrows represent the direction of the vertical axes (**c**); (**e**) 3D anatomical model of the PFM corresponding to the transverse section of the pubic muscle (cyan); (**f**) 2D regional mapping diagram based on the 3D anatomical model; (**g**) distribution map of PFM 24-regions based on physiology, the black arrows represent the pairing of different electrode units; (**h**) double-layer 3D contour-surface topographic map based on multi-electrode unit parameters. The lower layer displays functional parameters such as muscle contraction force, and the upper layer displays deep-level information on muscle contribution.

The MPDS is based on the precise signal acquisition of the ASEA. As shown in Figure 1a,b, the ASEA is an airbag-type inflatable PFM probe based on physiology, with a main body being a low modulus inflatable airbag (0.1~1.5 MPa). Multiple stretchable serpentine flexure electrode arrays are integrated on the surface of the airbag. This excellent adaptive structure allows it to utilize its unique inflatable capability to achieve a stable contact interface and can also suppress or reduce motion artifacts caused by internal environmental moisture, muscle movement, etc., thereby improving signal quality and achieving stable sEMG acquisition [28,29].

The ASEA matches the optimal acquisition sites of PFM multiple regions through anatomical and physiological principles and 3D extension control. Based on the dynamic movement trajectory of the muscles and the differential distribution of muscle fiber directions, electrode pairs are configured and formed into arrays, ensuring that each acquisition point can make contact with the target muscle belly region and maintain the relative stability of the electrode pair positions [30,31,32,33]. With the assistance of Tomography Ultrasound Imaging (TUI) technology in Figure 1c, we divide the muscle into several equidistant planes at the overall muscle level to confirm the location of muscle intersections as shown in Figure 1d. Moreover, Derive the distribution direction of muscle fibers in the corresponding planes. And then convert all positional information into 3D coordinate format. As shown in Figure 1e, build a 3D anatomical model of the muscle by integrating all image planes together. As shown in Figure 1f, the 3D coordinate points are mapped onto the 2D plane of the inflatable airbag device, with each 2D coordinate point serving as the location of the acquisition point. Based on the designed electrode positions, electrode patches are installed on the inflatable airbag device, and the accuracy of the positions is verified and adjusted under ultrasound imaging, resulting in an electrode array that can accurately locate regionalized muscles.

As shown in Figure 1g, the ASEA probe integrates a total of 32 electrode units, dividing them into 24 muscle detection areas, which are divided into 6 muscle groups, all of which are closely related to PFD [6,7], namely: iliococcygeus muscle (IC), pubococcygeus muscle (PC), puborectalis muscle (PR), vaginal sphincter (VS), urethral sphincter (US), and external anal sphincter (EAS). More detailed information about ASEA can be found in our previous work [25,26].

After obtaining the sEMG data from all channels, we will present all the muscle functions in the form of a double-layer 3D contour-surface topographic map (as shown in Figure 1h). Doctors can intuitively evaluate the sEMG parameter results related to the function of slow-twitch and fast-twitch muscles based on the color of the contour map on the lower layer and judge the abnormal functional areas. The height of the peaks on the upper layer of the topographic map can analyze the degree of muscle contribution, showing the differences between the abnormal functional areas and the surrounding muscle areas, and combining the sEMG parameters for lesion analysis.

### 2.2. MPDS System Overview

**Figure 2:**
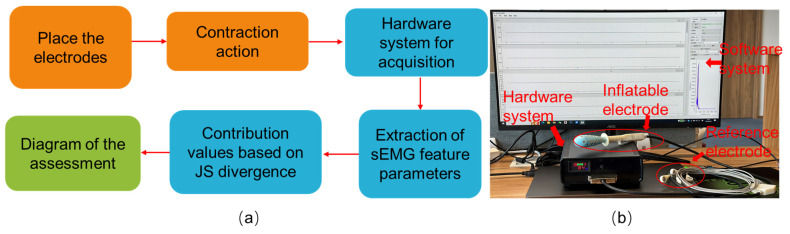
The system architecture of the MPDS. (**a**) Regionalized precise assessment process of the MPDS (**b**) Physical structure diagram of the MPDS.

Based on the research theory mentioned above, we designed the overall framework of the MPDS, as shown in Figure 2a. The MPDS is divided into three parts: the electrode terminal, the hardware acquisition system, and the software system. The workflow of the system is as follows: according to the site map of the PFM divided by regions, use the ASEA probe and reference electrode in the electrode terminal to place them on the corresponding acquisition points of the participants, and guide the participants to complete the standard contraction action. Further, use the hardware acquisition system to collect and process the sEMG data of the participant during the contraction action and complete anti-aliasing filtering and amplification processing. The key performance parameters of the hardware system are as follows: The sampling rate ranges from 1 K to 16 K samples per second, the resolution is 0.268 μV, the bandwidth is from 0 to 6.8 kHz, and the common mode rejection ratio is −110 dB. The processed data are transmitted wirelessly to the software system, where sEMG data feature extraction is performed. The calculated sEMG feature data are used as inputs for the regionalized sEMG functional feature assessment, and the assessment results are used as a reference to complete the muscle contribution assessment based on the JS algorithm. Meanwhile, the assessment results are displayed in real-time on the upper computer interface. Based on this system architecture, we have developed a multifunctional regional PFM functional assessment system. The physical description of the functional assessment system equipment is shown in Figure 2b.

### 2.3. Participants

We conducted a controlled clinical trial to validate the feasibility of applying our system clinically, specifically for the assessment of regional pelvic floor muscle function. The trial took place from February 2022 to February 2023 and included patients who were postpartum and attended the Department of Obstetrics and Gynecology at the Affiliated Hospital of Zhejiang University School of Medicine, as well as asymptomatic volunteers recruited through posters. Inclusion criteria were women of childbearing age, aged 18 or above, who had engaged in sexual intercourse and were able to tolerate vaginal examination. Exclusion criteria comprised pregnancy, acute inflammation of the urinary system, pelvic organs, or vagina, lower urinary tract obstruction, kidney disease, vaginal bleeding, pelvic malignant tumors, a history of pelvic radiation therapy, neurological disorders significantly affecting muscle function, and surgical history related to urinary incontinence or pelvic organ prolapse. Ultimately, a total of 129 women who were postpartum enrolled in the trial, divided into two groups based on the presence or absence of PSUI: the postpartum asymptomatic control group (Group A, *n* = 73) and the PSUI group (Group B, N = 56). The diagnostic criteria for PSUI included the occurrence of involuntary urine leakage during coughing, sneezing, or lifting heavy objects within one year postpartum. All participants received proper PFM contraction training and provided informed consent. We tabulated the demographic characteristics of both groups, as presented in Table 1, demonstrating no statistically significant differences between the two groups.

The general characteristics of the postpartum asymptomatic control group and the PSUI group are summarized in Table 1. The postpartum asymptomatic control group (Group A) comprised 73 participants, while the PSUI group (Group B) consisted of 56 participants.

The clinical trials in this work have been approved by the Ethics Committee of the Women’s Hospital, School of Medicine, Zhejiang University, Hangzhou, China (ID No. 067 (2019)), and the study was conducted in accordance with the ethical principles of the Declaration of Helsinki. All participants provided written and informed consent. Each participant was informed of the protocol prior to the trial and signed an informed consent form.

### 2.4. Clinical Trial Procedure

The clinical trial procedure is described as follows:

1. The participant is positioned supine, with the knees flexed, abducted, and externally rotated at 45°;
2. The ASEA is inserted into the participant’s vagina along a specified trajectory, and the balloon is inflated to ensure close contact between the electrode and the vaginal wall. Meanwhile, a reference electrode is placed on the participant’s right anterior superior iliac spine;
3. Before testing begins, the clinician instructs the participant on how to perform a proper contraction. After instruction, the participant is asked to follow the standardized Glazer assessment protocol as prompted by the device’s voice guidance;
4. Once testing is complete, the balloon is deflated and the vaginal electrode is removed. The used vaginal ASEA is disinfected and discarded;
5. The sEMG from the participants is processed by the MPDS. Initially obtain sEMG characteristic parameters for assessing the function of slow-twitch and fast-twitch pelvic floor muscles, as well as the identification of abnormal pelvic floor muscle sites. The JS algorithm is then used to evaluate muscle involvement, ultimately confirming the identification of abnormal pelvic floor muscle sites;
6. Finally, the conclusions of this study are assessed for reasonableness by combining clinical manifestations with pelvic floor electromyographic characteristics. This provides a basis for developing highly targeted intervention strategies focused on specific pelvic floor muscle sites in future work.

### 2.5. The sEMG Functional Feature Assessment Regionally

We used sEMG parameters related to tension, strength, fatigue, and coordination to assess the sustained contraction (slow-twitch muscles) and explosive contraction (fast-twitch muscles) as well as the function of the muscles in the PFM regions, and we visualized the differences in muscle function through color-coded 3D contour maps.

For the sustained contraction phase dominated by slow-twitch muscle, we chose the 10 s tonic contraction potential (TCP) to evaluate the strength intensity of slow-twitch muscles [34], which is defined as the average amplitude of the sEMG during five 10 s sustained contractions. A high TCP value indicates stronger slow-twitch muscle strength, while a low value indicates weaker muscle strength in that region. The ratio of endurance contraction (REC) was used to assess the fatigue of slow-twitch muscle [35], defined as the ratio of the average amplitude of the sEMG during the last 10 s of the sustained endurance contraction phase to the average amplitude during the first 10 s. A REC value closer to 1 indicates stronger resistance to fatigue, while a lower REC value suggests that the PFM may have entered a state of fatigue during the long-term contraction process. The variation of endurance contraction (VEC) was used to evaluate the stability of slow-twitch muscle during a 1 min sustained contraction, defined as the ratio of the standard deviation of the RMS amplitude during the 1 min sustained contraction process to the average value. For the rapid contraction phase dominated by fast-twitch muscles, we chose the rapid contraction rise time (RT) value to represent the muscle fatigue state during rapid contraction, defined as the time it takes for the muscle potential to rise from the resting state to the maximum during rapid contraction [36,37]. We selected the rapid contraction potential (RCP) to evaluate the strength intensity of fast-twitch muscles [35], defined as the average of the maximum RMS amplitude during five voluntary rapid contractions. A high RCP value indicates stronger fast-twitch muscle strength, while a low RCP value indicates weaker muscle strength in that region. The variability of rapid contractions (VRCs) was used to assess the stability of fast-twitch muscles, defined as the standard deviation of the amplitudes during five rapid contractions. A high VRCs indicates a decrease in the test participant’s ability to control the muscle and an inability to maintain stability during the contraction process. When the level of fatigue increases, the muscle spectrum will shift towards lower frequencies. Therefore, we chose the parameters related to median frequency (MF) to characterize muscle fatigue [38]. We analyzed and processed the MF data for 5 rapid contractions and 10s contractions performed 5 times for the PSUI group and the control group.

**Figure 3:**
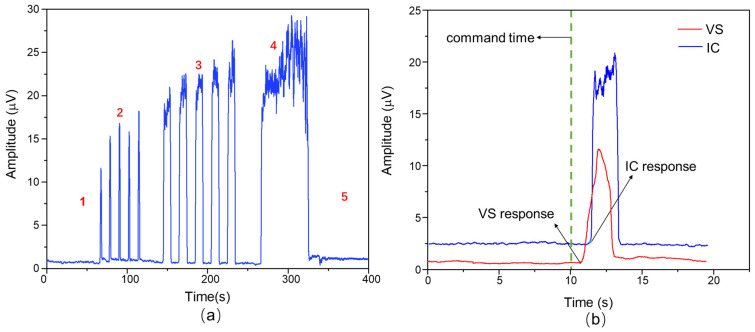
Electromyographic characteristic analysis. (**a**) sEMG waveform evaluated by Galzer. 1. Pre-Resting state (1 min); 2. rapid contraction (×5); 3. tonic contraction (×5); 4. endurance contraction (1 min); 5. post-resting state (1 min). (**b**) Schematic diagram of ATD in the VS and IC regions.

The focal muscle group functionality of the subjects can be identified, providing a reference for subsequent comprehensive assessments, through the aforementioned electromyographic characteristics. Figure 3a depicts the characteristic waveform graph after the subjects perform the standard Glazer assessment protocol [35]. The assessment actions are divided into five categories, with different contraction actions reflecting different emphasis on muscle functionality: (1) Pre-resting state for 1 min, primarily used to assess the subject’s muscle tension level. (2) Explosive rapid contractions, with a 10 s interval between each contraction, to evaluate the functional characteristics of fast-twitch muscles. This is repeated five times. (3) Sustained contractions for 10 s each, used to assess the functional characteristics of slow-twitch muscles. This is repeated five times with a 10 s interval between each contraction. (4) Single 1 min endurance contraction to evaluate the functional characteristics of slow-twitch muscles and fatigue level. (5) Post-resting state for 1 min. This stage, compared to the pre-resting state, helps observe different muscle characteristics before and after the actions.

Furthermore, by calculating the sequence of different muscle responses to commands in a region, the integrity of neural conduction in that area can be evaluated [39]. Firstly, note the time point of each command issued during each action, calculate the activation time of the muscles in the region during each rest-to-action process, and determine the ATD between the activation time and the action cue time. This provides the sequence of muscle contractions and assesses the degree of coordination of the muscles in the relevant area [40]. Finally, compare the parameter values of the subjects with the normal values of healthy individuals, identify the focal and synergistic muscles with coordination abnormalities under specific actions, and based on the subdivision of PFM collection areas, identify specific areas of dysfunction and reveal the regularity of muscle correlations. With an increase in the number of testers, group statistical comparisons are conducted to assess differences in functionality between groups and evaluate the degree of related functional abnormalities. Figure 3b illustrates the ATD schematic for the VS and IC regions.

### 2.6. The Muscle Contribution Assessment Based on JS Algorithm

**Figure 4:**
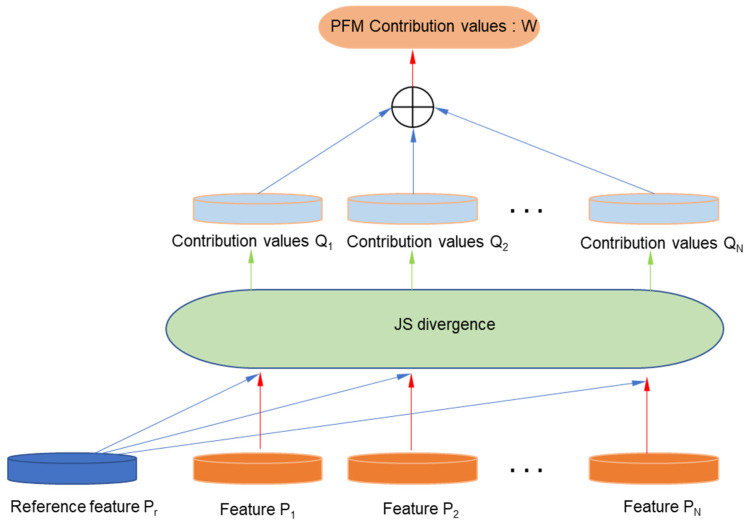
The implementation diagram of the PFM regional contribution assessment method based on JS divergence.

As shown in Figure 4, we calculated the contribution of each muscle region during contraction based on the Jensen–Shannon divergence, evaluated the differences in regional muscle compensation, and found the causes of abnormal states from the level of regional muscle coordination. Jensen–Shannon divergence is an improved metric designed to address the asymmetry of Kullback–Leibler (*KL*) divergence. *KL* divergence can be used to measure the difference between two distributions, and its calculation formula is as follows:

(1) $$KL\left(P\|Q\right)=\sum_{i=1}^{N}P\left(x_{i}\right)\log\frac{P\left(x_{i}\right)}{Q\left(x_{i}\right)}=\sum_{i=1}^{N}P\left(x_{i}\right)\left[\log P\left(x_{i}\right)-\log Q\left(x_{i}\right)\right]$$

In Formula (1), *P* and *Q* represent the feature parameters of two channels. According to the above formula, the difference between *P* and *Q* can be calculated. The calculation formula for Jensen–Shannon divergence is as follows:

(2) $$JS\left(P_{i},P_{j}\right)=\frac{1}{2}KL\left(P_{i}\|\frac{P_{i}+P_{j}}{2}\right)+\frac{1}{2}KL\left(P_{j}\|\frac{P_{i}+P_{j}}{2}\right)$$

In the Formula (2), i and j represent the channel numbers of the regions (from 1 to 24 for one parameter). We input the RCP, TCP, REC, RT, ATD, and other parameters mentioned above for each region. Accordingly, we obtain a feature contribution matrix $Q_{N\times C}$ based on Jensen–Shannon divergence, where N is the number of feature parameters, and each region channel has N mean-normalized Jensen–Shannon divergence values. The final Jensen–Shannon divergence values for each channel are obtained. Finally, we sum all the feature contribution values to obtain a matrix $W_{1\times C}$. The larger the W element value, the more information the acquisition region can obtain than other regions, indicating that the unit is more important. By comparing the contribution results of different regions W, we can gain a deeper understanding of the functional compensation phenomenon in the participant’s PFM and find the causes of abnormal states. Meanwhile, by jointly analyzing the basic sEMG parameters mentioned above, we can find the relationship between PFM function and PFD symptoms, laying the foundation for implementing a precise treatment plan.

### 2.7. Statistical Analysis

The data are reported as mean ± standard deviation. Depending on the distribution of the data, we employ unpaired samples *t*-test and Pearson’s chi-squared test to assess differences between the two sets of parameters. An asterisk (*) indicates statistical significance at (*p*) < 0.05. Error bars represent the 95% confidence interval. All data analyses were performed using MATLAB (Version 2020b) and SPSS (Version 25.0).

## 3. Results

### 3.1. Diagnosis Analysis of ATD

**Figure 5:**
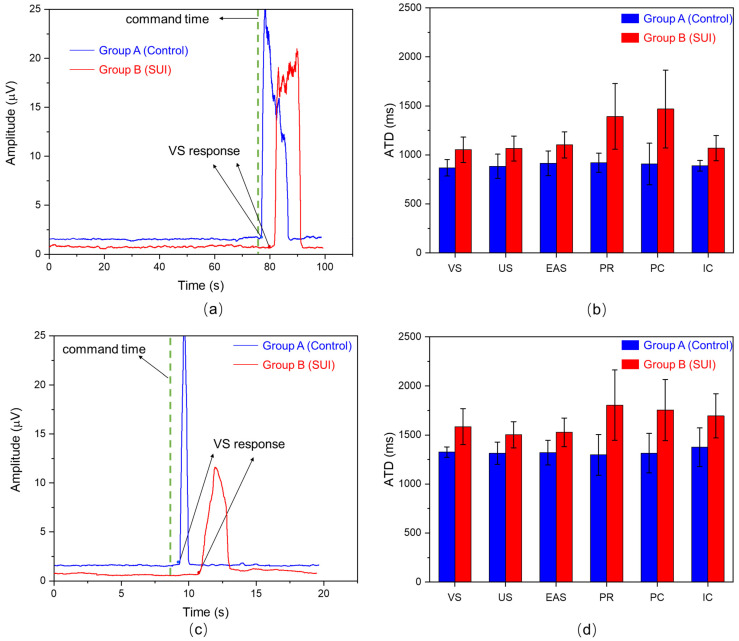
ATD Diagnosis Analysis of two groups. (**a**) ATD for slow-twitch muscles in participants from the PSUI group and the control group. The green dashed line represents the instruction time for initiating the action. (**b**) ATD values for slow-twitch muscles in 24 sets of collection points across 6 muscle regions for participants from both groups. The blue area represents Group A, the control group, while the red area represents Group B, the PSUI group. (**c**) ATD for fast-twitch muscles in participants from the PSUI group and the control group. The green dashed line represents the instruction time for initiating the action. (**d**) ATD values for fast-twitch muscles in 24 sets of collection points across 6 muscle regions for participants from both groups. The blue area represents Group A, the control group, while the red area represents Group B, the PSUI group.

As shown in Figure 5a, the schematic representation of ATD for slow-twitch muscles in the VS region of both groups during a 10 s sustained contraction. It is evident that the response time of the PSUI group is significantly longer compared to that of the control group. Figure 5b shows a bar chart comparing the ATD across the 6 muscle regions of the PFM during a 10 s sustained contraction for both groups. The overall mean ATD for Group A is 908.22 ms, and for Group B, it is 1088.09 ms. Overall, it is apparent that the ATD of the PSUI group is significantly longer than that of the control group throughout the entire PFM region. In different regions within their respective groups, the ATDs vary. The greatest difference between the two groups is observed between the PR and PC regions, with a difference in ATD between PRs of 473.31 ms and between PCs of 560.86 ms, representing a distribution higher than that of other regions by 158.64% and 177.52%, respectively. This indicates that these two regions respond much slower to instructions compared to the overall response, suggesting a significant issue of regional force coordination imbalance. Figure 5c shows the schematic representation of ATD for fast-twitch muscles in the VS region of both groups during rapid contractions. Similar to the sustained contraction scenario, the response time of the PSUI group is notably longer compared to that of the control group. As shown in Figure 5d, a bar chart comparing the ATD across the 6 muscle regions of the PFM during rapid contractions for both groups. Group A has an overall mean AT of 1319.35 ms, while Group B has an overall mean AT of 1580.93 ms. Similar to slow-twitch muscle, it is apparent that the ATD of the PSUI group is significantly longer than that of the control group throughout the entire PFM region. The largest differences between the two groups are observed between the PR and PC regions, with a difference in ATD between PRs of 505.87 ms and between PCs of 440.8 ms, representing a distribution higher than that of other regions by 169.68% and 134.04%, respectively. This also indicates a significant issue of force coordination imbalance in these two regions. In the subsequent detection using this system, special attention should be paid to these two abnormal regions to identify the underlying causes. Preliminary diagnostic analysis based on ATD reveals significant disparities in the response sequence of the entire PFM region between the two groups of participants, with the most notable differences occurring between the PSUI group and the control group in the PR and PC regions. ATD-based detection can provide diagnostic evidence and a basis for rapidly and accurately locating the degree of abnormality in PFM regions.

### 3.2. The Static Electromyographic Characteristics of Participants

Further analysis of the electromyographic signal characteristics extracted from participants in both groups allows for the assessment of abnormal states and their severity. We analyzed the features of the TCP and RCP electromyographic signals during 10 s sustained contractions and rapid contractions for both the PSUI group and the control group. As shown in Table 2, the results of TCP functional assessment for the PSUI group and the control group indicate significant differences in the PC (19.68 ± 16.13 vs. 11.54 ± 9.62, *p* = 0.001) and PR (19.58 ± 18.77 vs. 11.82 ± 10.06, *p* = 0.005) regions, suggesting that volunteers in Group A exhibit better fast-twitch muscle strength in these regions compared to those in Group B. As shown in Table 3, the results of RCP functional assessment for the PSUI group and the control group show significant differences in the VS (9.48 ± 4.67 vs. 24.45 ± 9.86, *p* < 0.001), PC (25.33 ± 19.33 vs. 16.04 ± 14.96, *p* = 0.004), and PR (25.26 ± 15.36 vs. 17.85 ± 12.17, *p* = 0.003) regions, indicating that volunteers in Group A exhibit better fast-twitch muscle strength in these regions compared to those in Group B. Furthermore, it was observed that the RCP value in the VS region for Group B was 166.67% higher than that for Group A. Subsequent detection in the system should focus on assessing the physiological characteristics of rapid contractions in the VS region for Group B, comparing and analyzing the high fast-twitch muscle strength in the VS region with the low muscle strength in the PC and PR regions.

Through this system, specific muscle strength characteristics of suspected abnormal regions can be analyzed, highlighting differences in muscle strength across various regions and accurately identifying the specific status of slow-twitch and fast-twitch muscles in the regions with abnormalities.

We analyzed and processed the MF data for five rapid contractions and 10 s contractions performed five times for the PSUI group and the control group. As shown in Table 4, the MF detection results for five rapid contractions of participants indicate significant differences in the PR (76.98 ± 2.04 vs. 34.00 ± 6.38, Cohen’s d = 9.074, *p* < 0.001) and PC (101.47 ± 8.65 vs. 63.01 ± 1.73, Cohen’s d = 6.166, *p* < 0.001) regions, suggesting that after five rapid contractions, the PSUI group’s muscles in the PR and PC regions are more fatigued compared to the control group. As shown in Table 5, the MF detection results for 10 s contractions performed five times reveal significant differences in the PR (82.90 ± 1.40 vs. 46.70 ± 11.91, Cohen’s d = 4.269, *p* < 0.001), PC (109.50 ± 2.10 vs. 71.43 ± 12.75, Cohen’s d = 4.167, *p* < 0.001), and VS (30.03 ± 5.35 vs. 72.26 ± 2.44, Cohen’s d = 10.157, *p* < 0.001) regions. This indicates that after five contractions of 10 s, the PSUI group’s muscles in the PR and PC regions are more fatigued compared to the control group, while the VS region shows the opposite, thereby assisting in verifying the results obtained in Table 3.

### 3.3. The Contribution Results Based on JS Algorithm

Based on the analysis of the differences in electromyographic characteristics between the two groups of volunteers, we identified different abnormal states and degrees of abnormalities in specific PFM regions within the PSUI group. Through contribution assessment based on the JS algorithm, we compared the contributions (W) of these different regions and their corresponding electromyographic characteristics to further validate the abnormal states of these regions. Additionally, we conducted anomaly analysis based on the differences in contributions between the two groups.

**Figure 6:**
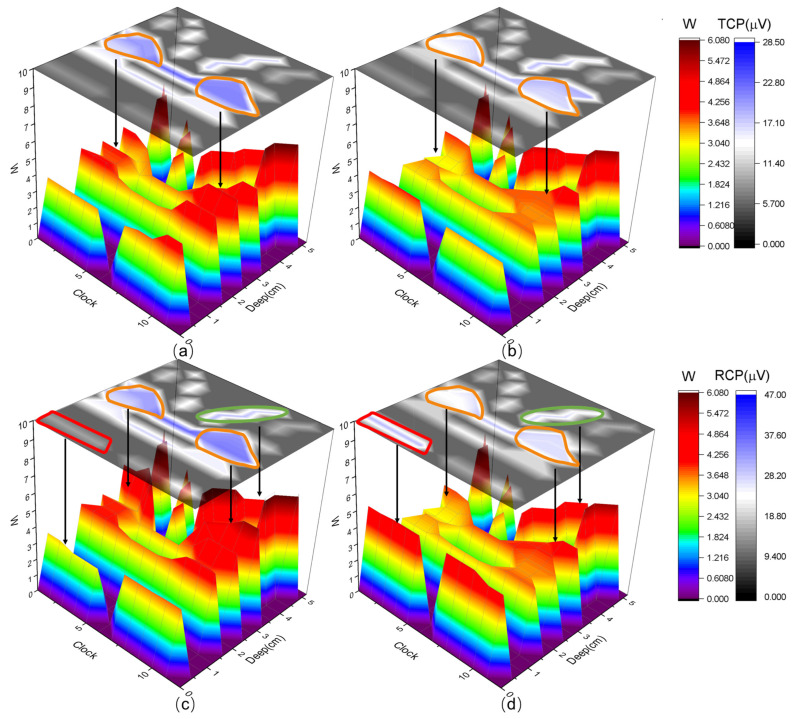
Slow-twitch and fast-twitch muscles contribution value evaluation of two groups. (**a**) For the control group and (**b**) PSUI group, the upper mapping plane represents the contour map of the TCP of slow-twitch, and the lower 3D surface represents the contribution value W of slow-twitch. Significant differences in parameters within the region can be observed. The yellow circle corresponds to the puborectalis muscle and the pubococcygeus muscle. It can be seen that in these two regions, the TCP of the upper plane and the contribution value W of the lower 3D surface exhibit similar trends. (**c**) For the control group and (**d**) the PSUI group, the upper mapping plane represents the contour map of the RCP of fast-twitch, and the lower 3D surface represents the contribution value W of fast-twitch. Significant differences in parameters within the region can be observed. The red circle corresponds to the pubovaginalis muscle, the yellow circle corresponds to the puborectalis muscle and the pubococcygeus muscle, and the green circle corresponds to the iliococcygeus muscle. It can be seen that in these three regions, the RCP of the upper plane and the contribution value W of the lower 3D surface exhibit similar trends.

Figure 6a,b depict the 3D surface mappings of the contribution (W) of slow-twitch muscles and electromyographic characteristics (RCP) in the PFM regions during testing for the PSUI group and the control group. We observed significantly higher RCP values in the PC and PR regions of the control group compared to the PSUI group, and the W contribution of the 3D surface was also significantly higher in the control group than in the PSUI group. The W contributions in the PC and PR regions of Group A were 52.34% and 49.38% higher than those of Group B, respectively.

Figure 6c,d depict the 3D surface mappings of the contribution (W) of fast-twitch muscles and electromyographic characteristics (RCP) in the PFM regions during testing for the PSUI group and the control group. We found significantly higher RCP values in the PC, PR, and IC regions of the control group compared to the PSUI group, and the W contribution of the 3D surface was also significantly higher in the control group than in the PSUI group. However, in the left VS region, the PSUI group exhibited significantly higher RCP values compared to the control group, and the W contribution of the 3D surface was also significantly higher in the PSUI group. The W contributions in the PC, PR, and IC regions of Group A were 26.70%, 54.19%, and 53.18% higher than those of Group B, respectively, while the left VS region of Group B was 152.38% higher than that of Group A.

## 4. Discussion

This study attempted for the first time to utilize a multi-region assessment system based on ASEA to accurately diagnose the areas of PFM dysfunction in the PSUI population. The results showed significant differences between the PSUI group and the control group in muscle response time, regional PFM function, and regional contribution. This system, through precise collection of sEMG signals using ASEA, achieved accurate localization and analysis of abnormal functional areas in PSUI, which cannot be achieved by existing technologies. In addition, compared to existing electrodes, ASEA collects data more objectively, and throughout the experiment, no subjects reported discomfort, pain, or other complaints, making it more conducive to full PFM contraction and fully reflecting the repeatability and safety of this technology.

In this study, for the first time, abnormal PFM areas in patients were rapidly located through the comparative use of ATD. The contraction time of ATD in both slow-twitch and fast-twitch muscles of the PSUI group was significantly longer than that in the control group, and this difference was statistically significant. This may be related to conduction abnormalities caused by damage to the pelvic floor nerves and the corresponding muscle groups during pregnancy and childbirth. Additionally, significant differences were found between the PR and PC regions in the two groups.

After locating the PFM areas, further analysis was conducted on the two types of PFM muscle strength in the PSUI group’s functionally abnormal regions. For slow-twitch PFM function, analysis of TCP static characteristic data indicated weaker slow-twitch muscle strength in the PC and PR regions of the PSUI group compared to the control group. For fast-twitch PFM function, analysis of RCP static characteristic data revealed a decrease in fast-twitch muscle strength in the VS, IC, PC, and PR regions of the PSUI group compared to the control group. Relevant studies have indicated that chronic compression or descent-related injuries to the PFM during pregnancy and childbirth can lead to postpartum PFM muscle strength decline, closely associated with the development of PSUI [41,42].

In the MPDS system, an innovative evaluation method utilizing regional muscle contribution and static characteristics of sEMG signals was employed to further validate the abnormal muscle regions in the PSUI group. In this study, we visualized the degree of PFM abnormalities using contour maps, showcasing them through color and height representation. For slow-twitch PFM function, the 3D surface mapping revealed significantly lower contributions (W) in the PC and PR regions of the PSUI group compared to the control group, with the upper-level mapping plane of TCP also significantly lower in the PSUI group. For fast-twitch PFM function, the 3D surface mapping showed significantly lower contributions (W) in the right PC, PR, and IC regions of the PSUI group compared to the control group, with the upper-level mapping plane of RCP also significantly lower in the PSUI group. However, in the left VS region, both the contribution (W) and RCP were significantly higher in the PSUI group. These results confirm the significant abnormalities observed in the PR and PC regions in ATD diagnosis, where PR and PC are crucial muscles for pelvic floor support, particularly the puborectalis muscle, a vital component. Damage to these muscles increases the compliance of the supporting structures below the urethra, leading to ineffective closure of the urethral lumen and subsequent PSUI [43]. Further analysis based on PFM contribution (W) revealed significantly stronger abnormalities in fast-twitch function in the VS region of the PSUI group compared to the control group. The VS muscle is involved in composing the middle layer of the PFM, including the urethrovaginal sphincter. Its increased function in PSUI may be related to compensatory contractions by other synergistic muscles to maintain urethral closure pressure in response to puborectalis muscle injury, providing insights into clinical pathophysiological mechanisms.

The above results indicate that, taking patients with PSUI as an example, this system not only reliably assesses PFM but also provides in-depth understanding of specific regional PFM dysfunction. It offers vital reference information and advanced therapeutic concepts for identifying causes in clinical practice. This underscores the potential of this system in clinical research and practice, providing a reliable foundation for future studies and clinical applications related to PFD.

## 5. Conclusions

In summary, this study collected pelvic floor sEMG data from women with PSUI and asymptomatic postpartum women using MPDS. We evaluated sEMG parameters, such as ATD, RCP, TCP, and MF, and identified significant abnormalities in the PC and PR regions. The comparison of regional contributions based on JS divergence and the 3D mapping of feature parameters further confirmed these significant abnormalities in the PR and PC regions. This system progressively locates abnormal areas, providing a new method for pinpointing localized PFM functional abnormalities that current technology cannot achieve, with the potential to improve diagnostic precision and reliability.

## Figures and Tables

**Table 1:** Baseline demographics of participants, postpartum and asymptomatic, control (Group A), and PSUI participants (Group B).

Variables	Group A N = 73	Group B N = 56	Effect Size (Cohen’s d)	*p*-Value
Age	29.1 (4.4)	30.3 (5.1)	0.279	0.154
BMI (kg/m^2^)	23.3 (3.1)	23.3 (4.2)	0	>0.99
Gravity			0.299	0.710
1	26 (35.6%)	16 (28.6%)		
2	26 (35.6%)	19 (33.9%)		
3	9 (12.3%)	12 (21.4%)		
4	8 (11.0%)	6 (10.7%)		
5	4 (5.5%)	3 (5.4%)		
Parity			0.311	0.170
1	43 (58.9%)	29 (51.8%)		
2	27 (37.0%)	27 (48.2%)		
3	3 (4.1%)	0 (0.0%)		

Note: Age and BMI were expressed as means ± standard deviations and parity and gravity were indicated as percentages. Abbreviations: BMI, body mass index.

**Table 2:** Slow-twitch muscle function assessment results of participants, postpartum and asymptomatic, control (Group A), and PSUI participants (Group B).

	Tonic Contraction Potential (TCP), μV			
Region	Group A N = 73	Group B N = 56	Effect Size (Cohen’s d)	*p*-Value
VS	4.33 ± 3.80	3.81 ± 3.95	0.134	0.452
US	22.89 ± 25.63	15.38 ± 12.60	0.372	0.046
EAS	21.25 ± 19.65	13.94 ± 10.81	0.461	0.014
PR	19.58 ± 18.77	11.82 ± 10.06	0.515	0.005
PC	19.68 ± 16.13	11.54 ± 9.62	0.613	0.001
IC	17.07 ± 16.45	13.64 ± 9.19	0.257	0.164

Note: TCP was expressed as tonic contraction potential. The data of Group A and Group B were expressed as means ± standard deviations. Abbreviations: VS, vaginal sphincter; US, urethral sphincter; EAS, external anal sphincter; PR, Puborectalis muscle; PC, Pubococcygeus muscle; IC, Iliococcygeus muscle.

**Table 3:** Fast-twitch muscle function assessment results of participants, postpartum and asymptomatic, control (Group A), and PSUI participants (Group B).

	Rapid Contraction Potential (RCP), μV			
Region	GROUP A N = 73	GROUP B N = 56	Effect Size (Cohen’s d)	*p*-Value
VS	9.48 ± 4.67	24.45 ± 9.86	1.940	<0.001
US	23.68 ± 19.34	22.13 ± 10.69	0.099	0.589
EAS	27.51 ± 18.06	22.62 ± 14.99	0.295	0.104
PR	25.26 ± 15.36	17.85 ± 12.17	0.535	0.003
PC	25.33 ± 19.33	16.04 ± 14.96	0.538	0.004
IC	16.41 ± 15.76	13.02 ± 11.75	0.244	0.181

Note: RCP was expressed as rapid contraction potential. The data of Group A and Group B were expressed as means ± standard deviations. Abbreviations: VS, vaginal sphincter; US, urethral sphincter; EAS, external anal sphincter; PR, Puborectalis muscle; PC, Pubococcygeus muscle; IC, Iliococcygeus muscle.

**Table 4:** MF (rapid contractions) assessment results of participants, postpartum and asymptomatic, control (Group A), and PSUI participants (Group B).

	MF (Rapid Contractions), Hz			
Region	Group A N = 73	Group B N = 56	Effect Size (Cohen’s d)	*p*-Value
VS	67.46 ± 3.92	67.65 ± 2.43	0.058	0.758
US	67.74 ± 1.85	64.61 ± 9.96	0.437	0.009
EAS	63.93 ± 2.06	56.13 ± 10.44	1.037	0.04
PR	76.98 ± 2.04	34.00 ± 6.38	9.074	<0.001
PC	101.47 ± 8.65	63.01 ± 1.73	6.166	<0.001
IC	89.39 ± 1.00	86.92 ± 12.72	0.274	0.101

Note: MF was expressed as the median frequency during rapid contractions. The data of Group A and Group B were expressed as means ± standard deviations. Abbreviations: VS, vaginal sphincter; US, urethral sphincter; EAS, external anal sphincter; PR, Puborectalis muscle; PC, Pubococcygeus muscle; IC, Iliococcygeus muscle.

**Table 5:** MF (10 s sustained contractions) muscle function assessment results of participants, postpartum and asymptomatic, control (Group A), and PSUI participants (Group B).

	MF (10 s Sustained Contractions), Hz			
Region	Group A N = 73	Group B N = 56	Effect Size (Cohen’s d)	*p*-Value
VS	30.03 ± 5.35	72.26 ± 2.44	10.157	<0.001
US	62.13 ± 1.70	61.32 ± 1.90	0.449	0.012
EAS	64.70 ± 10.83	60.83 ± 15.00	0.296	0.091
PR	82.90 ± 1.40	46.70 ± 11.91	4.269	<0.001
PC	109.50 ± 2.10	71.43 ± 12.75	4.167	<0.001
IC	80.22 ± 17.79	72.45 ± 36.32	0.272	0.113

Note: MF was expressed as the median frequency during 10 s sustained contractions. The data of Group A and Group B were expressed as means ± standard deviations. Abbreviations: VS, vaginal sphincter; US, urethral sphincter; EAS, external anal sphincter; PR, Puborectalis muscle; PC, Pubococcygeus muscle; IC, Iliococcygeus muscle.

## Data Availability

Data are available upon request to the authors.
